# 1154. Safety, Tolerability, and Viral Pharmacodynamics of the IgG Monoclonal Antibody Sotrovimab Administered via Intramuscular Injection for the Treatment of Early Mild-to-Moderate COVID-19

**DOI:** 10.1093/ofid/ofac492.992

**Published:** 2022-12-15

**Authors:** Anil K Gupta, Maria Teresa Perez-Rodríguez, Yaneicy Gonzalez-Rojas, Moti Ramgopal, Almena Free, Jennifer Han, Jennifer Moore, Rudrani Banerjee, Phillip Yates, Jill Walker, Gretja Schnell, Mary Beth Connolly, Andrea L Cathcart, Varsha Imber, Rabia Anselm, Lindsay Winograd, Nancy Haeusser, Scott Segal, Andrew Skingsley, Melissa Aldinger, Amanda Peppercorn, Jaynier Moya

**Affiliations:** Albion Finch Medical Centre, Toronto, Ontario, Canada; Hospital Álvaro Cunqueiro, Vigo, Pontevedra, Galicia sur Health Research Institute, Pontevedra, Galicia, Spain; Optimus U Corp, Miami, Florida; Midway Immunology and Research Center, Fort Pierce, FL; Pinnacle Research Group, LLC, Anniston, Alabama; GlaxoSmithKline, Collegeville, Pennsylvania; GlaxoSmithKline, Collegeville, Pennsylvania; GSK, Bangalore, Karnataka, India; GSK, Bangalore, Karnataka, India; GlaxoSmithKline, Collegeville, Pennsylvania; Vir Biotechnology, Inc., San Francisco, California; GSK, Bangalore, Karnataka, India; Vir Biotechnology, Inc., San Francisco, California; GSK, Bangalore, Karnataka, India; GSK, Bangalore, Karnataka, India; GSK, Bangalore, Karnataka, India; GSK, Bangalore, Karnataka, India; GSK, Bangalore, Karnataka, India; GlaxoSmithKline, Collegeville, Pennsylvania; Vir Biotechnology, Inc., San Francisco, California; GlaxoSmithKline, Collegeville, Pennsylvania; Pines Care Research Center LLC, Pembroke Pines, Florida

## Abstract

**Background:**

There is a continued need for therapeutics for the treatment of COVID-19, including intramuscular (IM) agents, which will enable broader use across a variety of healthcare delivery settings.

**Methods:**

COMET-PEAK (NCT04779879) is a 3-part study evaluating the safety, tolerability, pharmacokinetics (Part A), and viral pharmacodynamics (PD) of sotrovimab as treatment in adults ≥ 18 years with early mild/moderate COVID-19. In Parts B and C, the safety, tolerability and viral PD of sotrovimab administered as a 500 mg intravenous (IV) infusion or as a 500 mg or 250 mg IM injection, respectively, was evaluated. The primary objective for Parts B and C was to compare the virologic response of sotrovimab IM to IV, with an endpoint of mean area under the curve (AUC) of SARS-CoV-2 viral load as measured by qRT-PCR from Day 1 to Day 8 (AUC_D1-8_) in nasopharyngeal swabs and predefined 90% confidence interval (CI) limits of 0.5-2.0 indicating equivalence.

**Results:**

A total of 167 and 157 participants were enrolled in Part B and C, respectively, from February-July 2021. The median age of participants was 47 and 42 years in Part B and C, respectively, and ∼50% had ≥ 1 risk factor for progression to severe disease.

The viral load at baseline and through Day 29 of follow-up for each arm is shown in Table 1 and Figure 1. The primary objective was met for both study parts: the ratio of the least square geometric mean viral load AUC_(D1-8)_ of sotrovimab IM vs IV was 1.04 (90% CI, 0.98, 1.09) and 1.02 (90% CI, 0.94, 1.11), for Part B and C, respectively.

Through Day 29 of follow-up, the most common adverse event was injection site reactions (ISRs) in the IM arms. A total of 10 (12%) participants in the 500 mg IM group and 4 (5%) participants in the 250 mg IM group experienced an ISR, all Grade 1. Serious adverse events were uncommon, and related to COVID-19 progression, including one death in the 250 mg IM arm (Table 2). ISRs aside, there were few treatment-related AEs (2/84 IV, 1/82 IM) in Part B, none serious.

**Figure:**
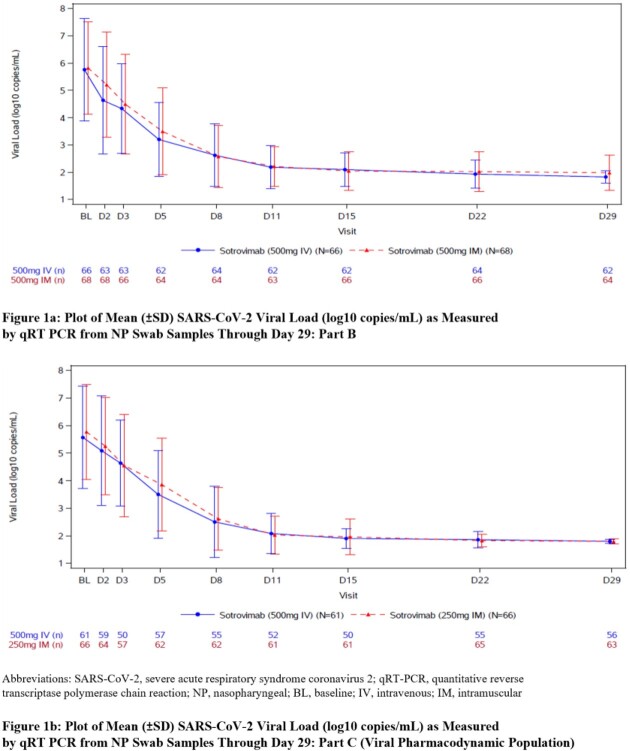

**Figure:**
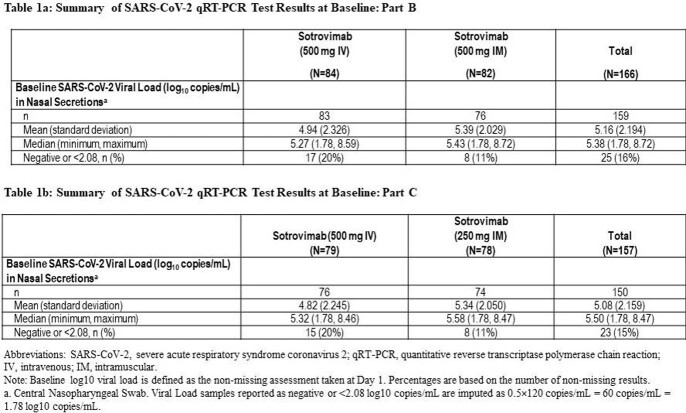

**Figure:**
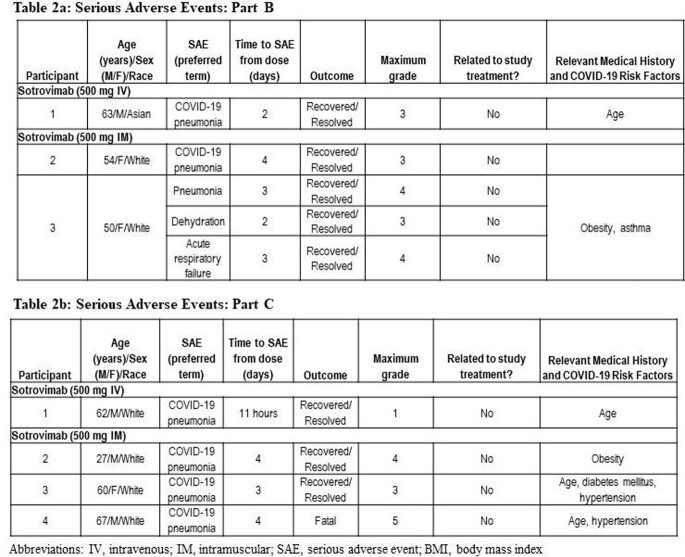

**Conclusion:**

IM administration of sotrovimab 500 mg and 250 mg each demonstrated equivalence to 500 mg sotrovimab IV in viral load assessments. Overall, there were no treatment-related serious AEs and sotrovimab was well tolerated. An 500 mg IM formulation will allow for expanded treatment potential with sotrovimab.

**Funding:**

Vir/GSK (NCT04779879).

**Disclosures:**

**Anil K. Gupta, MD**, Vir Biotechnology: Advisor/Consultant|Vir Biotechnology: Grant/Research Support|Vir Biotechnology: Speaker **Moti Ramgopal, MD, FACP, FIDSA**, AbbVie: Grant/Research Support|Gilead Sciences Inc.: Advisor/Consultant|Gilead Sciences Inc.: Grant/Research Support|Gilead Sciences Inc.: Honoraria|Gilead Sciences Inc.: Stocks/Bonds|GlaxoSmithKline: Advisor/Consultant|GlaxoSmithKline: Grant/Research Support|GlaxoSmithKline: Honoraria|GlaxoSmithKline: Stocks/Bonds|Janssen Research & Development LLC: Advisor/Consultant|Janssen Research & Development LLC: Grant/Research Support|Janssen Research & Development LLC: Honoraria|Janssen Research & Development LLC: Stocks/Bonds|Merck: Advisor/Consultant|Merck: Grant/Research Support|Merck: Honoraria|Merck: Stocks/Bonds|Shionogi: Grant/Research Support|ViiV: Advisor/Consultant|ViiV: Grant/Research Support **Jennifer Han, MD**, GlaxoSmithKline: Employee **Jennifer Moore, MD**, GlaxoSmithKline: Employee **Rudrani Banerjee, PhD**, GSK: Employee|GSK: Stocks/Bonds **Phillip Yates, PhD**, GSK: Employee during conduct of this research|GSK: Stocks/Bonds **Jill Walker, PhD**, GlaxoSmithKline: Employee **Gretja Schnell, PhD**, Vir Biotechnology: Employee|Vir Biotechnology: Stocks/Bonds **Mary Beth Connolly, PharmD**, GSK: Employee|GSK: Stocks/Bonds **Andrea L. Cathcart, PhD**, Vir Biotechnology: Employee|Vir Biotechnology: Stocks/Bonds **Varsha Imber, MSc**, GSK: Employee|GSK: Stocks/Bonds **Rabia Anselm, n/a**, GSK: Employee|GSK: Stocks/Bonds **Lindsay Winograd, MSc**, GSK: Employee|GSK: Stocks/Bonds **Nancy Haeusser, n/a**, GSK: Employee|GSK: Stocks/Bonds **Scott Segal, MD**, GSK: Employee|GSK: Stocks/Bonds **Andrew Skingsley, MD**, GlaxoSmithKline: Employee|GlaxoSmithKline: Stocks/Bonds **Melissa Aldinger, PharmD**, Vir Biotechnology: Employee|Vir Biotechnology: Stocks/Bonds **Amanda Peppercorn, MD**, GlaxoSmithKline: Employee|GlaxoSmithKline: Stocks/Bonds.

